# Enhancement of Chiroptical Signals by Circular Differential Mie Scattering of Nanoparticles

**DOI:** 10.1038/srep14463

**Published:** 2015-09-25

**Authors:** SeokJae Yoo, Q-Han Park

**Affiliations:** 1 Department of Physics, Korea University, Seoul, 136-713, Korea

## Abstract

We enhance the weak optical signals of small chiral molecules via circular differential Mie scattering (CDMS) of nanoparticles immersed in them. CDMS is the preferential Mie scattering of left- and right-handed circularly polarized light by nanoparticles whose sizes are about the same as the wavelength of light. Solving the Mie scattering theory for chiral media, we find that the CDMS signal of the particle is linearly proportional to the chirality parameter ***κ*** of the molecules. This linear amplitude enhancement by CDMS of the particle holds, even for large particles, which have a retardation effect. We also demonstrate that the CDMS of a nanoparticle is sensitive to changes of molecular concentration, and that the nanoparticle can be utilized as a chiroptical biosensor detecting the concentration of analyte. We expect that the enhancement of molecular chiroptical signals by CDMS will pave the way for novel chiroptical spectroscopy using nanostructures.

Chirality, which is a property of objects that cannot be superimposed on their mirror images, is a common feature of life’s building blocks such as actin, myosin, proteins, lipids, amino acids and sugars^1^. Measurement of molecular chiroptical effects, such as optical rotatory dispersion (ORD) and circular dichroism (CD), is used to obtain the stereochemical information of chiral molecules^2^. ORD is the optical rotation of linearly polarized light and CD is the extinction difference between left and right polarized light passing through the same molecular sample. However, small molecules, being much smaller than the wavelength of light, have inherently weak chiroptical signals which are challenging to detect. Measurement of the chiroptical signals of small molecules is thus limited to samples in microgram quantities^3^. To overcome this drawback of chiroptical signals as indicators of stereochemical information, it has been noted that nanostructures^3^,^4^,^5^,^6^ can amplify the differential absorption by molecules of oppositely polarized circular light via the molecule-plasmon Coulomb interaction^7^ and optical chirality enhancement^8^. However, these approaches are still in the Rayleigh regime, in that the molecules are still small compared with the wavelength *λ* of the absorbed or scattered light. In the Rayleigh scattering regime, area (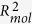)-normalized energy absorbed and scattered by molecules of radius *R*_*mol*_ is proportional to *R*_*mol*_/*λ* and (*R*_*mol*_/*λ*)^4^ respectively, and the small molecular size fundamentally restricts absorbed energy to the molecular scale factor *R*_*mol*_/*λ* resulting in negligible scattered energy^9^,^10^,^11^.

Our approach, in contrast, uses larger achiral nanoparticles to enhance molecular chiroptical signals through circular differential scattering which is in the Mie regime since the sizes of the nanoparticles are comparable to the wavelength of light. Generalizing the Mie scattering theory for chiral molecular media, we show that achiral nanoparticles embedded in chiral molecules exhibit circular differential scattering in which the intensities of the scattering of left- and right-handed circularly polarized incident light are different. This circular differential Mie scattering (CDMS) by nanoparticles acts as a carrier signal that can amplify the signals of chiral molecules. As nanoparticles in the Mie regime are large, their CDMS can significantly amplify the chiroptical signals of other molecules. We also demonstrate that the CDMS of plasmonic and high refractive index nanoparticles perform when used to sense molecular concentration changes. Furthermore, our work enables the real-time and local measurement of molecular dynamics, such as protein binding kinetics, in the vicinity of the nanoparticles using CDMS.

## Results

### Chiral Mie scattering theory

Media composed of chiral molecules are described by the constitutive relation^12^


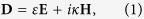



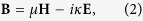


where *ε*, *μ*, and ***κ*** are the electric permittivity, magnetic permeability, and chirality parameters of the chiral molecule medium, respectively. The chirality parameter ***κ*** of the chiral molecule medium represents the chiroptical signals of the chiral molecules. Electromagnetic fields propagating through chiral media display circular birefringence^12^; left- and right-handed circularly polarized light experience different speeds through the medium according to wavevectors 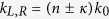 with the conventional refractive index of the medium *n* and vacuum wavevector 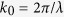. The subscript *L* (*R*) corresponds to a plus (minus) sign in left-handed (right-handed) circular polarization with wavevectors *k*_*L*_ (*k*_*R*_).

**Figure 1:**
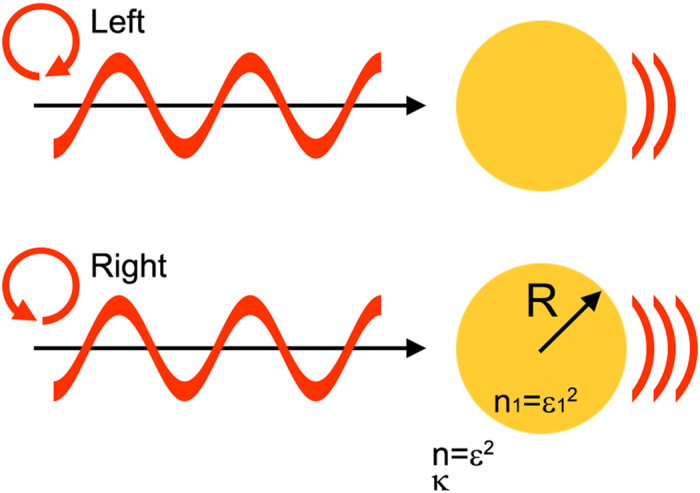
Schematic drawing of CDMS. Nanoparticles with refractive index 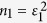 and radius *R* are embedded in the surrounding chiral molecule medium with refractive index *n* = *ε*^2^ and chirality parameter ***κ***. Nanoparticles embedded in the chiral molecule medium display CDMS according to the polarization of the incident waves.

We study the CDMS of nanoparticles embedded in a chiral molecule medium through solving the problem of full-wave Mie scattering in chiral media. Figure 1 is a schematic of the CDMS process. Here, we assume that the chirality parameter ***κ*** of chiral media is purely real because most chiral molecules do not absorb in the visible spectral region. To solve the Mie problem for chiral media, we break down the incident, scattered, and internal fields of spherical particles in terms of the circular basis set 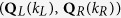 according to the wavevector *k*_*L*,*R*_^9^. Scattered fields **E**_*s*_ are decomposed into


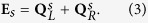


The circular bases for the scattered fields **E**_*s*_ are given by


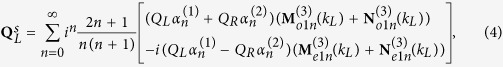



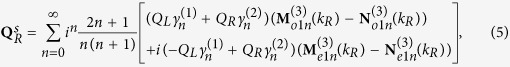


with scattering coefficients 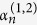 and 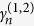, vector spherical harmonics **M** and **N**^9^, and polarization amplitudes of the incident field *Q*_*L*,*R*_. For left- and right-handed circularly polarized incident light, the sets of polarization amplitudes are (*Q*_*L*_, *Q*_*R*_) = (1, 0) and (*Q*_*L*_, *Q*_*R*_) = (0, 1), respectively. The boundary conditions of the total electromagnetic fields at the surface of the sphere determine the expansion coefficients of the scattered and internal fields (see Supplementary information for a full derivation of the chiral Mie scattering theory). We also calculate the rates of extinction of the incident light energy. Incident light energy is scattered or absorbed by a particle, and the scattering or absorption cross sections are the rates of those processes normalized by incident light intensity. The extinction, scattering and absorption cross sections of an achiral sphere embedded in a chiral molecule medium are given by


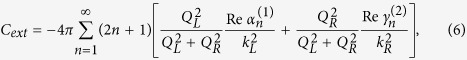



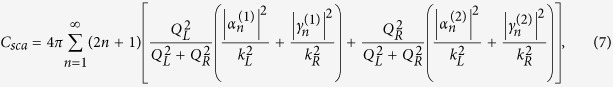



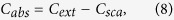


with resonance order *n* = 1, 2, ···.

### Circular differential Rayleigh scattering of nanoparticles

Before covering the circular differential scattering of nanoparticles in the Mie regime, we first consider small nanoparticles in the Rayleigh regime. The complex mathematical forms of Eqs (6, 7, 8) may hinder understanding the physics of circular differential scattering. In the Rayleigh scattering regime (*nk*_0_*R* ≪ 1), the polynomial expansion of the scattering coefficients 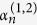 and 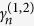 can provide more physically revealing expressions. For dipole order *n* = 1, the polynomial expansion of the scattering and absorption cross sections, Eqs (7 and 8), about *k*_0_*R* to the lowest order give


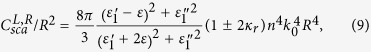



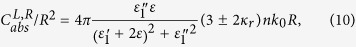


where the relative chirality parameter of the surrounding medium is 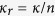, and the particle radius is *R*. The complex permittivity of the particle is represented by 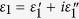. The superscript *L* (*R*) in the cross sections *C*_*sca*,*abs*_ corresponds to plus (minus) signs in Eqs (9 and 10). The circular differential scattering and absorption cross sections of the nanoparticle are written as


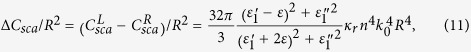



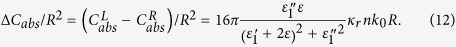


We can identify a few key trends of the circular differential Rayleigh scattering and absorption of a particle embedded in a chiral molecule medium. As for conventional Rayleigh scattering, circular differential Rayleigh scattering and absorption cross sections of a particle, Eqs (11 and 12), are respectively proportional to (*R/λ*)^4^ and *R/λ*. Circular differential Rayleigh scattering also displays localized surface plasmon resonance (LSPR) of electric dipole order when the dispersive dielectric function 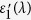 of the particle meets the LSPR condition 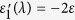. Importantly, circular differential scattering and absorption is linearly proportional to the chirality parameter ***κ*** of the surrounding medium. This gives rise to linear amplitude enhancement of the molecular chiroptical signals by circular differential scattering in the Rayleigh scattering regime.

### Circular differential Mie scattering of nanoparticles

**Figure 2:**
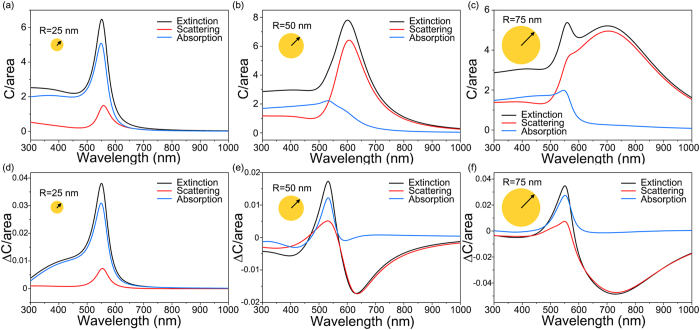
(**a**–**c**) Extinction (black), scattering (blue), and absorption cross section per particle area (red) of gold nanoparticles with radii *R* = 25, 50, and 75 nm embedded in chiral molecule medium of refractive index *n* = 1.5 and chirality parameter ***κ*** = 0.01. (**d**–**f**) Corresponding circular differential extinction (black), scattering (blue), and absorption cross section per particle area (red) of the same gold nanoparticles.

Equipped with conclusions from the quasi-static expressions, we now study the properties of circular differential scattering of large nanoparticles in the Mie scattering regime. By full-wave chiral Mie calculation, we will confirm whether large nanoparticles can modulate chirality parameter ***κ*** of the surrounding medium in the Mie regime as they do in the Rayleigh regime. Figure 1 shows spectra of the extinction cross section *C*_*ext*_, scattering cross section *C*_*sca*_, and absorption cross section *C*_*abs*_ and the corresponding circular differential cross sections Δ*C*_*ext*_, Δ*C*_*sca*_, and Δ*C*_*abs*_ of gold nanoparticles with different radii. The optical constant of gold is taken from tabulated literature data^13^. The refractive index *n* and chirality parameter ***κ*** of the surrounding chiral molecule medium are respectively 1.5 and 0.01 which are typical values for chiral liquids in the visible spectral region^14^,^15^. We note that refractive index and chirality parameter of the surrounding medium can be dispersive. Chiral liquids such as limonene and carvone are weakly dispersive so that our assumption of negligible imaginary parts of *n* and ***κ*** are still valid^16^ (see Supplementary Information for the calculation of CDMS in the weakly dispersive chiral medium). The most important feature of Fig. 2 is that the order of the circular differential scattering cross section Δ*C*_*sca*_ is readily measurable by conventional spectroscopy techniques^10^.

In Fig. 2a,d, the small nanoparticle of *R* = 25 nm belongs to the Rayleigh scattering regime, and thus its scattering characteristics follow Eqs (9, 10, 11, 12). In contrast, in Fig. 2b–f the larger nanoparticles scattering characteristics follow the Mie regime. In Fig. 2b,e (*R* = 50 nm) and Fig. 2c,f (*R* = 75 nm), the peak of dipolar LSPR shifts to red as the gold nanoparticles become larger. Interestingly, in Fig. 2e,f, dipolar LSPRs in circular differential cross section also exhibit a red shift, but their signs are reversed and become negative. Also, the scattering cross sections of larger nanoparticles surpass absorption cross sections, as shown in Fig. 2b,e (*R* = 50 nm) and Fig. 2c,f (*R* = 75 nm). In summary, we find the distinguishing characteristics of CDMS to be the negative dips of the circular differential scattering cross section and the dominance of scattering, both of which are absent in Rayleigh scattering.

### Linear amplitude enhancement of chiroptical signals by CDMS

**Figure 3:**
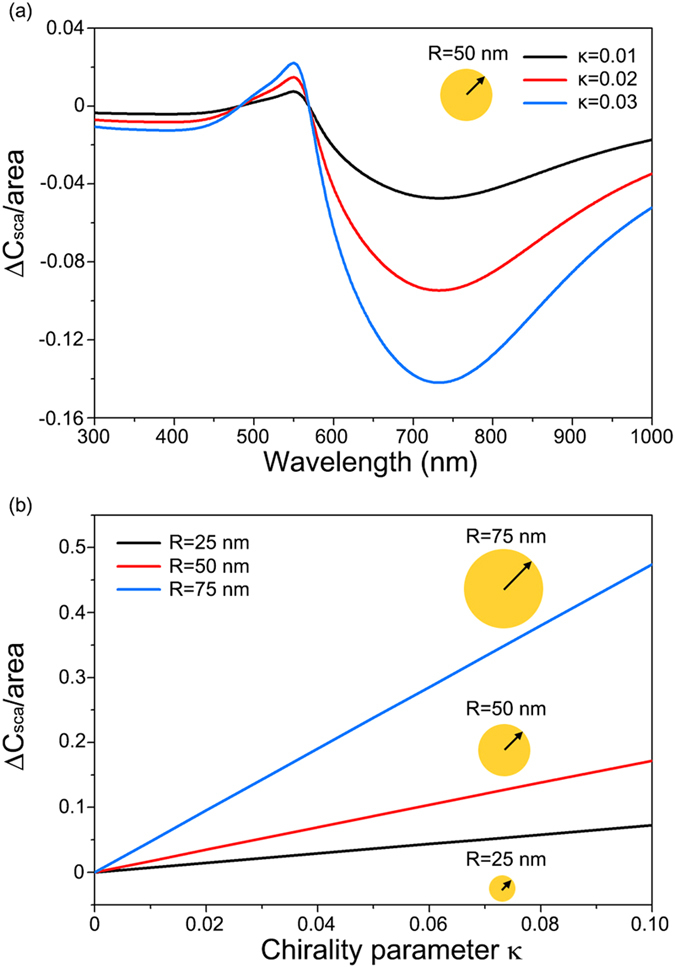
(**a**) Circular differential scattering cross section per particle area of gold nanoparticles with radius *R* = 50 nm embedded in chiral molecule medium of refractive index *n* = 1.5 and chirality parameter ***κ*** = 0.01 (black), 0.02 (red), and 0.03 (blue). (**b**) Change in maximum circular differential scattering cross section per particle area of a gold nanoparticles with radii *R* = 25, 50, and 75 nm embedded in chiral molecule medium of refractive index *n* = 1.5 and varying chirality parameter ***κ*** from 0 to 0.1.

Scattering by nanoparticles enhances the amplitude of the molecular chiroptical signal. As shown in Eq. (11), the chirality parameter ***κ*** of background molecules is directly multiplied according to the scattering lineshape of nanoparticles in the Rayleigh regime. Even for larger particles, the amplitude enhancement of the chiroptical signal ***κ*** by CDMS was found to be linear. As shown in Fig. 3a, the Δ*C*_*sca*_ of gold nanoparticles of *R* = 50 nm, embedded in molecules of refractive index *n* = 1.5 and varying ***κ***, increases with the chirality parameter ***κ*** of the surrounding medium, without significant spectral shift of the plasmon resonances. It is also notable that in Fig. 3a the Δ*C*_*sca*_ spectra of the chirality parameter ***κ*** = 0.02 and 0.03 are exactly double and triple, respectively, the Δ*C*_*sca*_ spectrum of ***κ*** = 0.01. To take a closer look at this linear amplitude modulation of the surrounding medium chirality parameter ***κ*** on the Mie scattering, in Fig. 3b we plotted the maximum differential scattering cross sections Δ*C*_*sca*_ of three gold nanoparticles of different radii (*R* = 25 nm, 50 nm, and 70 nm). Figure 3b confirms that the circular differential scattering cross sections Δ*C*_*sca*_ of large gold nanoparticles are linearly proportional to the chirality parameter ***κ*** in the range to ***κ*** = 0.1. The chirality parameter ***κ*** is inherently weak for chiral molecules in nature. This inherent weakness of ***κ*** results in the linear ***κ***-dependence in CDMS because ***κ***-independent term drops out in Δ*C* and the higher-order terms are negligible. The linear ***κ***-dependence is also independent of the size of particle, and thus this holds even for large nanoparticles including the retardation effect.

### Chiral molecule sensing by the amplitude enhancement on CDMS

**Figure 4:**
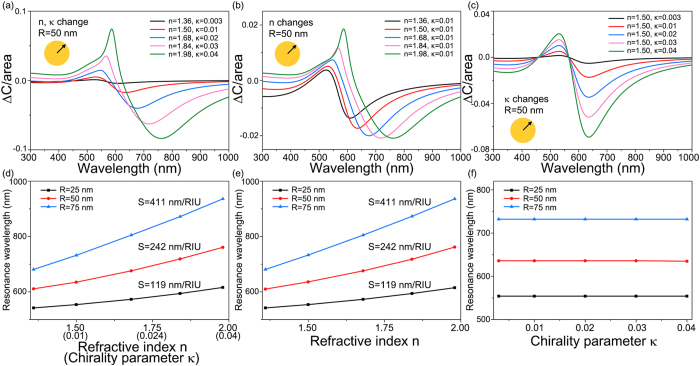
Change in lineshapes of circular differential scattering cross section per particle area of a gold nanoparticle with radius *R *= 50 nm corresponding to (**a**) simultaneous change in *n* and *κ*, (**b**) change in *n* with fixed *κ*= 0.01, and (**c**) change in *κ* with fixed *n *= 1.5. Sensitivities of gold nanoparticles with radii *R* = 25, 50, and 75 nm corresponding to (**d**) simultaneous changes in n and *κ*, (**e**) change in n with fixed ***κ*** = 0.01, and (**f**) change in ***κ*** with fixed *n* = 1.5.

In Fig. 4a, we study the spectral change of the circular differential scattering cross section according to the concentration of chiral molecules near gold nanoparticles of *R* = 50 nm. We assume that the permittivity, refractive index, and chirality parameter of the chiral molecule medium are written as 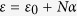, 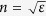, and ***κ*** = *NG*, respectively, with *ε*_0_ = the permittivity of aqueous solvent = 1.3^2^ = 1.69, *N* = number density of molecule, *α* = electric polarizability of the molecule, and *G *= mixed electric-magnetic polarizability of the molecule. We estimate the magnitude of polarizabilities to be *Nα* = 0.56 and *G* = 0.018*α*, and this estimation of polarizabilities provides reasonable parameters of *n* = 1.5 and ***κ*** = 0.01 for typical chiral liquid samples. In Fig. 4, we increase the number density of the molecule *N* in order to study the resulting change in spectral CDMS. Figure 4a shows that the magnitude of the circular differential scattering cross section Δ*C*_*sca*_ increases with the red shift of the LSPR position, as the number density of molecule *N* is increased up to six times. Note that this change corresponds to a change in the refractive index and chirality parameter from *n* = 1.5 and ***κ*** = 0.01 to *n* = 2.25 and ***κ*** = 0.06. Figure 4b, is a plot of change in resonant wavelength in circular differential scattering cross section against change in the number density of molecule *N*. Figure 4d shows that resonant wavelengths of gold nanoparticles tend to increase linearly with the refractive index of the medium. Sensitivities 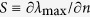 of gold nanoparticles with radius *R* = 25 nm, 50 nm, and 70 nm are respectively 119 nm/RIU, 242 nm/RIU and 411 nm/RIU, where RIU denotes the refractive index unit. Note that typical sensitivity of conventional plasmonic biosensors for non-chiral molecules are the order of 10^2^ nm/RIU, and sensitivities of gold nanoparticles in Fig. 4d are comparable to the conventional plasmonic biosensors^17^. The sensitivity of the spectral change of circular differential scattering in Fig. 4d indicates that real time CD measurement can be realistically applied to the study of the kinetics of molecules near nanostructures^18^,^19^.

To understand the effect of changes in refractive index *n* and chirality parameter ***κ*** of the surrounding medium to spectral changes shown in Fig. 4a,d, we separately vary refractive index *n* and chirality parameter ***κ*** of the surrounding medium. In Fig. 4b,e, we vary refractive index *n* with fixed chirality parameter ***κ*** = 0.01. Resonance dips of the circular differential scattering cross section Δ*C*_*sca*_ shifts to red wavelengths in Fig. 4b. In Fig. 4c,f, we vary chirality parameter ***κ*** with fixed refractive index *n* = 1.5. We find resonance dips of Δ*C*_*sca*_ do not move, but their magnitudes become larger negative values by increasing chirality parameter ***κ*** in Fig. 4c. Figure 4f shows resonance wavelengths of gold nanoparticles with various radii do not shifted by chirality parameter ***κ***. From Fig. 4b–f, we conclude that shifts of resonance wavelength is originated from changes in refractive index *n*, while changes in magnitude of Δ*C*_*sca*_ come from changes in chirality parameter ***κ***.

### CDMS of dielectric nanoparticles

**Figure 5:**
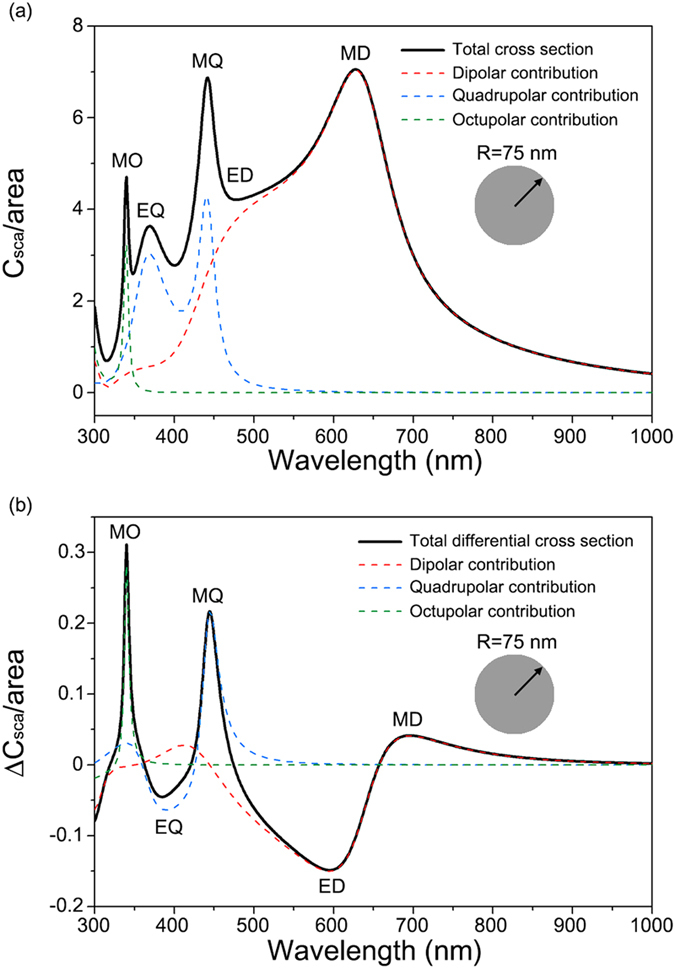
(**a**) Scattering cross section of a silicon nanoparticle per particle area with radius *R *= 75 nm embedded in chiral molecule medium of refractive index *n* = 1.5 and chirality parameter ***κ*** = 0.01. The solid line (black) is the total scattering cross sections, and dashed red, blue, and green lines respectively are the dipolar, quadrupolar, and octupolar contributions to the total scattering cross section. MD (magnetic dipole), ED (electric dipole), MQ (magnetic quadrupole), EQ (electric quadrupole), MO (magnetic octupole) are denoted in the plot. (**b**) The corresponding circular differential scattering cross section per particle area of the silicon nanoparticle.

CDMS is not limited to plasmonic nanostructures. In fact, recent research into dielectric nanoparticles and metamaterials have demonstrated that the scattering of high-index dielectric nanostructures are also measurable^20^,^21^,^22^,^23^,^24^,^25^,^26^. Figure 5 plots the circular differential scattering of a silicon nanoparticle of refractive index *n*_*Si*_ = 4 and radius *R* = 75 nm embedded in chiral molecule medium of refractive index *n* = 1.5 and chirality parameter ***κ*** = 0.01. Figure 5a plots the spectrum of the total cross section of the silicon nanoparticle. In Fig. 5a, the silicon nanoparticle has multiple resonances such as a magnetic dipole (MD), electric dipole (ED), magnetic quadrupole (MQ), electric quadrupole (EQ), and magnetic octupole (MO) according to the hierarchy of resonance in high-index dielectric nanoparticles^20^. The differential scattering cross section Δ*C*_*sca*_ in Fig. 5b inherits multiple electric and magnetic resonances of the scattering cross section *C*_*sca*_. In Fig. 5b, the electric resonances (ED and EQ) show negative signals in differential scattering cross section, the same as for gold nanoparticles, while the magnetic resonances (MD, MQ, and MO) show positive ones. Compared to plasmonic nanoparticles of the same size (*R* = 75 nm) in Fig. 2c,f, the silicon nanoparticles exhibit a differential cross section up to approximately 10 times larger due to the lossless nature of dielectric materials. In addition to the magnitude of Δ*C*_*sca*_, higher order resonances with smaller linewidths such as quadrupoles and octupoles are also accessible in the dielectric nanoparticles.

## Discussion

Our theory of CDMS resolves the mismatch between the experimental results and theoretical predictions of chiral field generation. According to recent reports, experimental CD measurements are much stronger than theoretical expectations of chiral field generation^3^,^14^,^27^. In the theory of chiral field generation in ref. 8absorption of a chiral molecule is given by 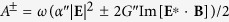, where *α*″ and *G*″ are the imaginary parts of the electric polarizability and the isotropic mixed electric-magnetic dipole polarizability, respectively. The differential absorption of the molecule is 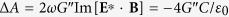 with the optical chirality 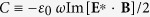. From this differential absorption Δ*A*, many researchers have attempted to enhance the optical chirality *C* using nanostructures^3^,^4^,^5^,^6^,^14^,^28^,^29^,^33^. However, the enhancement of optical chirality using nanostructures suggested theoretically is significantly smaller than the enhancements obtained experimentally. The main reason for this mismatch comes from the size of the molecule. Area (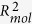)-normalized differential absorption Δ*A* of the molecule is proportional to *R*_*mol*_/*λ* because the isotropic mixed electric-magnetic dipole polarizability *G*″ is proportional to molecular volume 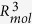
30. This is consistent with Rayleigh scattering. In contrast, CDMS cross sections of nanoparticles are proportional to (*R*_*mol*_/*λ*)^4^, as in Eq. (11). Consequently, the amplified CD signals of large nanoparticles observed in experiments may principally originate from circular differential scattering. This argument would explain the mismatch between the experimentally obtained and theoretically predicted results.

We find that the CDMS cross sections of particles embedded in a chiral molecule medium, Eqs (7 and 11) are proportional to resonance strength. That is, we can expect that the close-packing of plasmonic nanoparticles can improve the circular differential scattering cross section because it increases the resonance strength of the nanoparticles^31^. On the other hand, it has been recently discovered that gold nanorods immersed in structurally chiral cellulose nanocrystals displays strong polarization sensitive response in experiments^32^. We expect that CDMS of various shaped nonchiral nanoparticles, such as nanorods and nanoplates, can be applied to chiroptical spectroscopy. In the future, we will extend our research into the CDMS of closely packed nanoparticle systems and other shaped nonchiral nanoparticles.

## Additional Information

**How to cite this article**: Yoo, S.J. and Park, Q.-H. Enhancement of Chiroptical Signals by Circular Differential Mie Scattering of Nanoparticles. *Sci. Rep.* **5**, 14463; doi: 10.1038/srep14463 (2015).

## Supplementary Material

Supplementary Information
