# Supplementary material for: Enhancement of Chiroptical Signals by Circular Differential Mie Scattering of Nanoparticles
Source: Sci Rep. 2015 Sep 25;5:14463. doi: 10.1038/srep14463 (PMC4585887; doi:10.1038/srep14463)
Supplement: Supplementary Information [file srep14463-s1.doc]

Supplementary Information:

Enhancement of Chiroptical Signals by Circular Differential Mie Scattering of Nanoparticles

*SeokJae Yoo and Q-Han Park^,*^*

Department of Physics, Korea University, Seoul, 136-713, Korea.

1. **Derivation of the chiral Mie scattering theory**
   1. **Wavefield decomposition of electromagnetic fields**

Constitutive relation of chiral molecule media is

$\mathbf{D}=\varepsilon\mathbf{E}+i\kappa\mathbf{H}$,

$\mathbf{B}=\mu\mathbf{H}-i\kappa\mathbf{E}$,

where $\varepsilon$, $\mu$, and $\kappa$ are the electric permittivity, magnetic permeability, and chirality parameter of the chiral molecule medium, respectively. Combining constitutive relations (Eq. and ) and Maxwell’s curl equations, we obtain

$\left(\begin{matrix} \nabla\times\mathbf{E} \\ \nabla\times\mathbf{H} \end{matrix}\right)=\left(\begin{matrix} k_{0}\kappa& ik_{0}\mu\\ -ik_{0}\varepsilon& k_{0}\kappa\end{matrix}\right)\left(\begin{matrix} \mathbf{E} \\ \mathbf{H} \end{matrix}\right)$,

where $k_{0}$ is the vacuum wavenumber. Eq. can be diagonalized by

$\left(\begin{matrix} \nabla\times\mathbf{Q}_{L} \\ \nabla\times\mathbf{Q}_{R} \end{matrix}\right)=\left(\begin{matrix} k_{0}\left(n+\kappa\right) & 0 \\ 0 & -k_{0}\left(n-\kappa\right) \end{matrix}\right)\left(\begin{matrix} \mathbf{Q}_{L} \\ \mathbf{Q}_{R} \end{matrix}\right)=\left(\begin{matrix} k_{L} & 0 \\ 0 & -k_{R} \end{matrix}\right)\left(\begin{matrix} \mathbf{Q}_{L} \\ \mathbf{Q}_{R} \end{matrix}\right)$,

through the linear transformation of the set of electromagnetic fields $\left(\mathbf{E},\mathbf{H}\right)$ to the set of wavefields $\left(\mathbf{Q}_{L},\mathbf{Q}_{R}\right)$. The linear transformation of electromagnetic fields is given by

$\left(\begin{matrix} \mathbf{E} \\ \mathbf{H} \end{matrix}\right)=\left(\begin{matrix} 1 & 1 \\ -i/\eta& i/\eta\end{matrix}\right)\left(\begin{matrix} \mathbf{Q}_{L} \\ \mathbf{Q}_{R} \end{matrix}\right)$.

Wavefields $\mathbf{Q}_{L}$ and $\mathbf{Q}_{R}$ possess wavevector eigenvalues $k_{L}=\left(n+\kappa\right)k_{0}$ and $k_{R}=\left(n-\kappa\right)k_{0}$, respectively.

In this work, we first consider the scattering problem of a sphere with radius $a$, refractive index $n_{1}$, the electric permittivity $\varepsilon_{1}=n_{1}^{2}$, the magnetic permeability $\mu_{0}$, and the chirality parameter $\kappa_{1}$ embedded in the surrounding chiral molecule medium with refractive index $n$, the electric permittivity $\varepsilon=n^{2}$, the magnetic permeability $\mu_{0}$, and the chirality parameter $\kappa$. This leads to *the solution for scattering of light by the chiral sphere in the chiral medium*.

We can express the incident, scattered, and internal electromagnetic fields in terms of the wavefields $\left(\mathbf{Q}_{L},\mathbf{Q}_{R}\right)$. The incident fields are written as

$\begin{matrix} \mathbf{Q}_{L}^{i}=Q_{L}\left(\hat{\mathbf{x}}+i\hat{\mathbf{y}}\right) \\ =\sum_{n=0}^{\infty} Q_{L}i^{n}\frac{2n+1}{n(n+1)}\left[\left(\mathbf{M}_{o1n}^{\left(1\right)}\left(k_{L}\right)+\mathbf{N}_{o1n}^{\left(1\right)}\left(k_{L}\right)\right)-i\left(\mathbf{M}_{e1n}^{\left(1\right)}\left(k_{L}\right)+\mathbf{N}_{e1n}^{\left(1\right)}\left(k_{L}\right)\right)\right] \end{matrix}$,

$\begin{matrix} \mathbf{Q}_{R}^{i}=Q_{R}\left(\hat{\mathbf{x}}-i\hat{\mathbf{y}}\right) \\ =\sum_{n=0}^{\infty} Q_{R}i^{n}\frac{2n+1}{n(n+1)}\left[\left(\mathbf{M}_{o1n}^{\left(1\right)}\left(k_{R}\right)-\mathbf{N}_{o1n}^{\left(1\right)}\left(k_{R}\right)\right)+i\left(\mathbf{M}_{e1n}^{\left(1\right)}\left(k_{R}\right)-\mathbf{N}_{e1n}^{\left(1\right)}\left(k_{R}\right)\right)\right] \end{matrix}$.

The scattered fields are

$\mathbf{Q}_{L}^{s}=\sum_{n=0}^{\infty} i^{n}\frac{2n+1}{n(n+1)}\left[a_{n}\left(\mathbf{M}_{o1n}^{\left(3\right)}\left(k_{L}\right)+\mathbf{N}_{o1n}^{\left(3\right)}\left(k_{L}\right)\right)-ib_{n}\left(\mathbf{M}_{e1n}^{\left(3\right)}\left(k_{L}\right)+\mathbf{N}_{e1n}^{\left(3\right)}\left(k_{L}\right)\right)\right]$,

$\mathbf{Q}_{R}^{s}=\sum_{n=0}^{\infty} i^{n}\frac{2n+1}{n(n+1)}\left[c_{n}\left(\mathbf{M}_{o1n}^{\left(3\right)}\left(k_{R}\right)-\mathbf{N}_{o1n}^{\left(3\right)}\left(k_{R}\right)\right)+id_{n}\left(\mathbf{M}_{e1n}^{\left(3\right)}\left(k_{R}\right)-\mathbf{N}_{e1n}^{\left(3\right)}\left(k_{R}\right)\right)\right]$.

The internal fields of the sphere are

$\mathbf{Q}_{L}^{1}=\sum_{n=0}^{\infty} i^{n}\frac{2n+1}{n(n+1)}\left[f_{on}\left(\mathbf{M}_{o1n}^{\left(1\right)}\left(k_{L1}\right)+\mathbf{N}_{o1n}^{\left(1\right)}\left(k_{L1}\right)\right)-if_{en}\left(\mathbf{M}_{e1n}^{\left(1\right)}\left(k_{L1}\right)+\mathbf{N}_{e1n}^{\left(1\right)}\left(k_{L1}\right)\right)\right]$,

$\mathbf{Q}_{R}^{1}=\sum_{n=0}^{\infty} i^{n}\frac{2n+1}{n(n+1)}\left[g_{on}\left(\mathbf{M}_{o1n}^{\left(1\right)}\left(k_{R1}\right)-\mathbf{N}_{o1n}^{\left(1\right)}\left(k_{R1}\right)\right)+ig_{en}\left(\mathbf{M}_{e1n}^{\left(1\right)}\left(k_{R1}\right)-\mathbf{N}_{e1n}^{\left(1\right)}\left(k_{R1}\right)\right)\right]$.

$a_{n}$, $b_{n}$, $c_{n}$, $d_{n}$, $f_{on}$, $f_{en}$, $g_{on}$, and $g_{en}$ are the expansion coefficients for the scattered and internal fields. $\mathbf{M}_{omn}$, $\mathbf{M}_{emn}$, $\mathbf{N}_{omn}$ and $\mathbf{N}_{emn}$ are the vector spherical harmonics of order $m$ and $n$. The subscripts *o* and *e* of vector spherical harmonics denote odd and even, respectively. The expanded form of the incident fields dictate the form of the spherical harmonics expansion, and thus the order of expansion $m$ is fixed to $m=1$^1,2^. Vector spherical harmonics are given by

,

,

where $z_{n}$ is any of the spherical Bessel functions: the spherical Bessel functions $j_{n}$, the spherical Neumann functions $y_{n}$, the spherical Hankel functions of the first kind $h_{n}^{\left(1\right)}$, and the spherical Hankel functions of the second kind $h_{n}^{\left(2\right)}$. For as $z_{n}$, superscript 1 of $\mathbf{M}^{\left(1\right)}$ and $\mathbf{N}^{\left(1\right)}$ corresponds to spherical Bessel functions $j_{n}$, and superscript 2 of $\mathbf{M}^{\left(2\right)}$ and $\mathbf{N}^{\left(2\right)}$ corresponds to spherical Hankel functions of the first kind $h_{n}^{\left(1\right)}$. The angle-dependent functions are defined as^1^

$\pi_{n}=\frac{P_{n}^{1}}{\sin\theta}$,

$\tau_{n}=\frac{dP_{n}^{1}}{d\theta}$,

with the associated Legendre polynomials .

- 1. **Boundary conditions and matrix equations for scattering coefficients**

Expansion coefficients ($a_{n}$, $b_{n}$, $c_{n}$, $d_{n}$, $f_{on}$, $f_{en}$, $g_{on}$, and $g_{en}$) for the scattered and internal fields are determined by the boundary conditions at the surface of the sphere. The boundary conditions are given by

$E_{i\theta}+E_{s\theta}=E_{1\theta}$,

,

$H_{i\theta}+H_{s\theta}=H_{1\theta}$,

.

Using the boundary conditions, we obtain two matrix equations for the expansion coefficients. Matrix equations are given by

$\mathbf{M}_{1}\left(\begin{matrix} a_{n} \\ c_{n} \\ f_{en} \\ g_{en} \end{matrix}\right)=\mathbf{M}_{2}$,

$\mathbf{M}_{3}\left(\begin{matrix} b_{n} \\ d_{n} \\ f_{on} \\ g_{on} \end{matrix}\right)=\mathbf{M}_{4}$.

Matrices are written as

$\mathbf{M}_{1}=\left(\begin{matrix} h_{n}^{\left(1\right)}\left(k_{L}a\right) & h_{n}^{\left(1\right)}\left(k_{R}a\right) & -j_{n}\left(k_{L1}a\right) & -j_{n}\left(k_{R1}a\right) \\ \frac{\left[k_{L}ah_{n}^{\left(1\right)}\left(k_{L}a\right)\right]^{'}}{k_{L}a} & -\frac{\left[k_{R}ah_{n}^{\left(1\right)}\left(k_{R}a\right)\right]^{'}}{k_{R}a} & -\frac{\left[k_{L1}aj_{n}\left(k_{L1}a\right)\right]^{'}}{k_{L1}a} & \frac{\left[k_{R1}aj_{n}\left(k_{R1}a\right)\right]^{'}}{k_{R1}a} \\ h_{n}^{\left(1\right)}\left(k_{L}a\right) & -h_{n}^{\left(1\right)}\left(k_{R}a\right) & -mj_{n}\left(k_{L1}a\right) & mj_{n}\left(k_{R1}a\right) \\ \frac{\left[k_{L}ah_{n}^{\left(1\right)}\left(k_{L}a\right)\right]^{'}}{k_{L}a} & \frac{\left[k_{R}ah_{n}^{\left(1\right)}\left(k_{R}a\right)\right]^{'}}{k_{R}a} & -m\frac{\left[k_{L1}aj_{n}\left(k_{L1}a\right)\right]^{'}}{k_{L1}a} & -m\frac{\left[k_{R1}aj_{n}\left(k_{R1}a\right)\right]^{'}}{k_{R1}a} \end{matrix}\right)$,

$\mathbf{M}_{2}=\left(\begin{matrix} -Q_{L}j_{n}\left(k_{L}a\right)-Q_{R}j_{n}\left(k_{R}a\right) \\ -Q_{L}\frac{\left[k_{L}aj_{n}\left(k_{L}a\right)\right]^{'}}{k_{L}a}+Q_{R}\frac{\left[k_{R}aj_{n}\left(k_{R}a\right)\right]^{'}}{k_{R}a} \\ -Q_{L}j_{n}\left(k_{L}a\right)+Q_{R}j_{n}\left(k_{R}a\right) \\ -Q_{L}\frac{\left[k_{L}aj_{n}\left(k_{L}a\right)\right]^{'}}{k_{L}a}-Q_{R}\frac{\left[k_{R}aj_{n}\left(k_{R}a\right)\right]^{'}}{k_{R}a} \end{matrix}\right)$,

$\mathbf{M}_{3}=\left(\begin{matrix} h_{n}^{\left(1\right)}\left(k_{L}a\right) & -h_{n}^{\left(1\right)}\left(k_{R}a\right) & j_{n}\left(k_{L1}a\right) & -j_{n}\left(k_{R1}a\right) \\ -\frac{\left[k_{L}ah_{n}^{\left(1\right)}\left(k_{L}a\right)\right]^{'}}{k_{L}a} & -\frac{\left[k_{R}ah_{n}^{\left(1\right)}\left(k_{R}a\right)\right]^{'}}{k_{R}a} & -\frac{\left[k_{L1}aj_{n}\left(k_{L1}a\right)\right]^{'}}{k_{L1}a} & -\frac{\left[k_{R1}aj_{n}\left(k_{R1}a\right)\right]^{'}}{k_{R1}a} \\ h_{n}^{\left(1\right)}\left(k_{L}a\right) & h_{n}^{\left(1\right)}\left(k_{R}a\right) & -mj_{n}\left(k_{L1}a\right) & -mj_{n}\left(k_{R1}a\right) \\ -\frac{\left[k_{L}ah_{n}^{\left(1\right)}\left(k_{L}a\right)\right]^{'}}{k_{L}a} & \frac{\left[k_{R}ah_{n}^{\left(1\right)}\left(k_{R}a\right)\right]^{'}}{k_{R}a} & m\frac{\left[k_{L1}aj_{n}\left(k_{L1}a\right)\right]^{'}}{k_{L1}a} & -m\frac{\left[k_{R1}aj_{n}\left(k_{R1}a\right)\right]^{'}}{k_{R1}a} \end{matrix}\right)$,

$\mathbf{M}_{4}=\left(\begin{matrix} -Q_{L}j_{n}\left(k_{L}a\right)+Q_{R}j_{n}\left(k_{R}a\right) \\ Q_{L}\frac{\left[k_{L}aj_{n}\left(k_{L}a\right)\right]^{'}}{k_{L}a}+Q_{R}\frac{\left[k_{R}aj_{n}\left(k_{R}a\right)\right]^{'}}{k_{R}a} \\ -Q_{L}j_{n}\left(k_{L}a\right)-Q_{R}j_{n}\left(k_{R}a\right) \\ Q_{L}\frac{\left[k_{L}aj_{n}\left(k_{L}a\right)\right]^{'}}{k_{L}a}-Q_{R}\frac{\left[k_{R}aj_{n}\left(k_{R}a\right)\right]^{'}}{k_{R}a} \end{matrix}\right)$,

with the wavevector $k_{L1,R1}=\left(n_{1}\pm\kappa_{1}\right)k_{0}$ inside the sphere. Solving two matrix equations, Eq. and , we find

$a_{n}=Q_{L}\alpha_{n}^{\left(1\right)}+Q_{R}\alpha_{n}^{\left(2\right)}$,

$b_{n}=Q_{L}\alpha_{n}^{\left(1\right)}-Q_{R}\alpha_{n}^{\left(2\right)}$,

$c_{n}=Q_{L}\gamma_{n}^{\left(1\right)}+Q_{R}\gamma_{n}^{\left(2\right)}$,

$d_{n}=-Q_{L}\gamma_{n}^{\left(1\right)}+Q_{R}\gamma_{n}^{\left(2\right)}$,

with the scattering coefficients $\alpha_{n}^{\left(1,2\right)}$ and $\gamma_{n}^{\left(1,2\right)}$. When the chirality parameter of the sphere turns off, i.e. $\kappa_{1}=0$, we can obtain the solution for *scattering of light by the achiral sphere in the chiral medium*. Explicitly, the scattering coefficients $\alpha_{n}^{\left(1,2\right)}$ and $\gamma_{n}^{\left(1,2\right)}$ of an achiral sphere ($\kappa_{1}=0$) are written by

$\alpha_{n}^{\left(1\right)}=\frac{1}{\Delta}\left[\begin{matrix} -2m\left(j_{n}\left(k_{1}a\right)^{2}\frac{\left[k_{L}aj_{n}\left(k_{L}a\right)\right]^{'}}{k_{L}a}\frac{\left[k_{R}ah_{n}^{\left(1\right)}\left(k_{R}a\right)\right]^{'}}{k_{R}a}+j_{n}\left(k_{L}a\right)h_{n}^{\left(1\right)}\left(k_{R}a\right)\frac{\left[k_{1}aj_{n}\left(k_{1}a\right)\right]^{'}}{k_{1}a}^{2}\right) \\ +\left(m^{2}+1\right)j_{n}\left(k_{1}a\right)\frac{\left[k_{1}aj_{n}\left(k_{1}a\right)\right]^{'}}{k_{1}a}\left(j_{n}\left(k_{L}a\right)\frac{\left[k_{R}ah_{n}^{\left(1\right)}\left(k_{R}a\right)\right]^{'}}{k_{R}a}+h_{n}^{\left(1\right)}\left(k_{R}a\right)\frac{\left[k_{L}aj_{n}\left(k_{L}a\right)\right]^{'}}{k_{L}a}\right) \end{matrix}\right]$,

$\alpha_{n}^{\left(2\right)}=\frac{1}{\Delta}\left[\left(m^{2}-1\right)j_{n}\left(k_{1}a\right)\frac{\left[k_{1}aj_{n}\left(k_{1}a\right)\right]^{'}}{k_{1}a}\left(j_{n}\left(k_{R}a\right)\frac{\left[k_{R}ah_{n}^{\left(1\right)}\left(k_{R}a\right)\right]^{'}}{k_{R}a}-h_{n}^{\left(1\right)}\left(k_{R}a\right)\frac{\left[k_{R}aj_{n}\left(k_{R}a\right)\right]^{'}}{k_{R}a}\right)\right]$,

$\gamma_{n}^{\left(1\right)}=\frac{1}{\Delta}\left[\left(m^{2}-1\right)j_{n}\left(k_{1}a\right)\frac{\left[k_{1}aj_{n}\left(k_{1}a\right)\right]^{'}}{k_{1}a}\left(j_{n}\left(k_{L}a\right)\frac{\left[k_{L}ah_{n}^{\left(1\right)}\left(k_{L}a\right)\right]^{'}}{k_{L}a}-h_{n}^{\left(1\right)}\left(k_{L}a\right)\frac{\left[k_{L}aj_{n}\left(k_{L}a\right)\right]^{'}}{k_{L}a}\right)\right]$,

$\gamma_{n}^{\left(2\right)}=\frac{1}{\Delta}\left[\begin{matrix} -2m\left(j_{n}\left(k_{1}a\right)^{2}\frac{\left[k_{R}aj_{n}\left(k_{R}a\right)\right]^{'}}{k_{R}a}\frac{\left[k_{L}ah_{n}^{\left(1\right)}\left(k_{L}a\right)\right]^{'}}{k_{L}a}+j_{n}\left(k_{R}a\right)h_{n}^{\left(1\right)}\left(k_{L}a\right)\frac{\left[k_{1}aj_{n}\left(k_{1}a\right)\right]^{'}}{k_{1}a}^{2}\right) \\ +\left(m^{2}+1\right)j_{n}\left(k_{1}a\right)\frac{\left[k_{1}aj_{n}\left(k_{1}a\right)\right]^{'}}{k_{1}a}\left(j_{n}\left(k_{R}a\right)\frac{\left[k_{L}ah_{n}^{\left(1\right)}\left(k_{L}a\right)\right]^{'}}{k_{L}a}+h_{n}^{\left(1\right)}\left(k_{L}a\right)\frac{\left[k_{R}aj_{n}\left(k_{R}a\right)\right]^{'}}{k_{R}a}\right) \end{matrix}\right]$,

where the denominator $\Delta$ is defined as

$\begin{matrix} \Delta=\frac{\left[k_{R}ah_{n}^{\left(1\right)}\left(k_{R}a\right)\right]^{'}}{k_{R}a}j_{n}\left(k_{1}a\right)\left(2m\frac{\left[k_{L}ah_{n}^{\left(1\right)}\left(k_{L}a\right)\right]^{'}}{k_{L}a}j_{n}\left(k_{1}a\right)-\left(m^{2}+1\right)\frac{\left[k_{1}aj_{n}\left(k_{1}a\right)\right]^{'}}{k_{1}a}h_{n}^{\left(1\right)}\left(k_{L}a\right)\right) \\ +\frac{\left[k_{1}aj_{n}\left(k_{1}a\right)\right]^{'}}{k_{1}a}h_{n}^{\left(1\right)}\left(k_{R}a\right)\left(2m\frac{\left[k_{1}aj_{n}\left(k_{1}a\right)\right]^{'}}{k_{1}a}h_{n}^{\left(1\right)}\left(k_{L}a\right)-\left(m^{2}+1\right)\frac{\left[k_{L}ah_{n}^{\left(1\right)}\left(k_{L}a\right)\right]^{'}}{k_{L}a}j_{n}\left(k_{1}a\right)\right) \end{matrix}$.

$m=n_{1}/n$ denotes relative refractive index of the sphere.

- 1. **Extinction, scattering, and absorption cross section**

Extinction and scattering cross sections are defined by

,

,

where $W_{ext}$ and $W_{sca}$ are the rate at which energy is extinct and scattered from the incident wave. $I_{in}=\left(Q_{L}^{2}+Q_{R}^{2}\right)/\eta$ is the intensity of the incident fields. By the orthogonality of the angle-dependent functions, we obtain the extinction, scattering, and absorption cross sections as follows:

,

,

.

- 1. **Radiation pattern of CDMS**

The asymptotic expressions of the first kind Hankel functions in the far-field region ($kr\gg1$) are given by

$$h_{n}^{\left(1\right)}\left(\rho\right)\sim\frac{\left(-i\right)^{n}e^{i\rho}}{i\rho}$$

$$\frac{dh_{n}^{\left(1\right)}\left(\rho\right)}{d\rho}\sim\frac{\left(-i\right)^{n}e^{i\rho}}{\rho}$$

The scattered fields in the far-field region are derived by substituting Eq. and into Eq. and . Then, polar and azimuthal components of the scattered fields are written as

,

,

,

.

The scattering direction $\hat{\mathbf{r}}$ and the forward direction $\hat{\mathbf{z}}$ define the scattering plane^1,2^. The scattered fields are expressed in terms of the unit vectors along the parallel direction $\hat{\mathbf{e}}_{\parallel}=\hat{\mathbf{\theta}}$ and the perpendicular direction $\hat{\mathbf{e}}_{\perp}=-\hat{\mathbf{\varphi}}$ to the scattering plane. Using the scattering plane, the scattered fields in the far-field region are

,

with $E_{s,\theta}=Q_{L,\theta}^{s}+Q_{R,\theta}^{s}$ and .


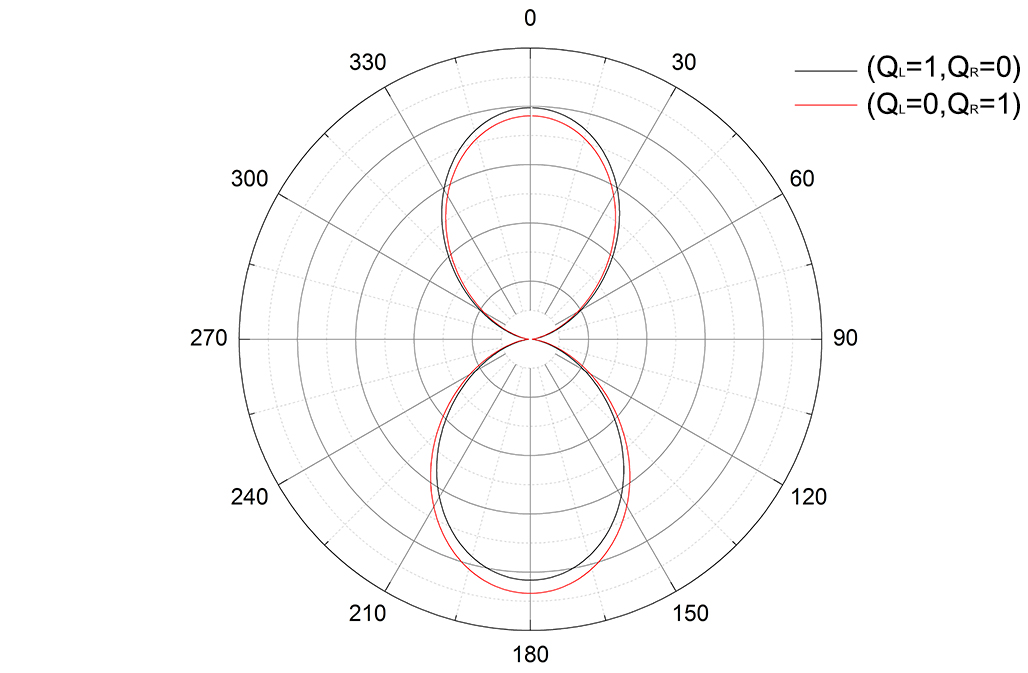


FIG. S1. Radiation pattern of a gold nanoparticle with radius $R$=75 nm, embedded in chiral molecule medium of refractive index $n$=1.5 and chirality parameter $\kappa$=0.01. Black line (red line) corresponds to the scattered field intensity parallel to the scattering plane with the left (right) circularly polarized incident light.

Fig. S1 shows radiation pattern of a gold nanoparticle with radius $R$=75 nm, embedded in chiral molecule medium of refractive index $n$=1.5 and chirality parameter $\kappa$=0.01. In Fig. S1, we find slight difference in forward and backward scattering intensity according to the handedness of the incident light. We conclude that this difference causes CDMS cross sections of nanoparticles immersed in chiral molecule medium.

1. **CDMS of Drude metal nanoparticle**


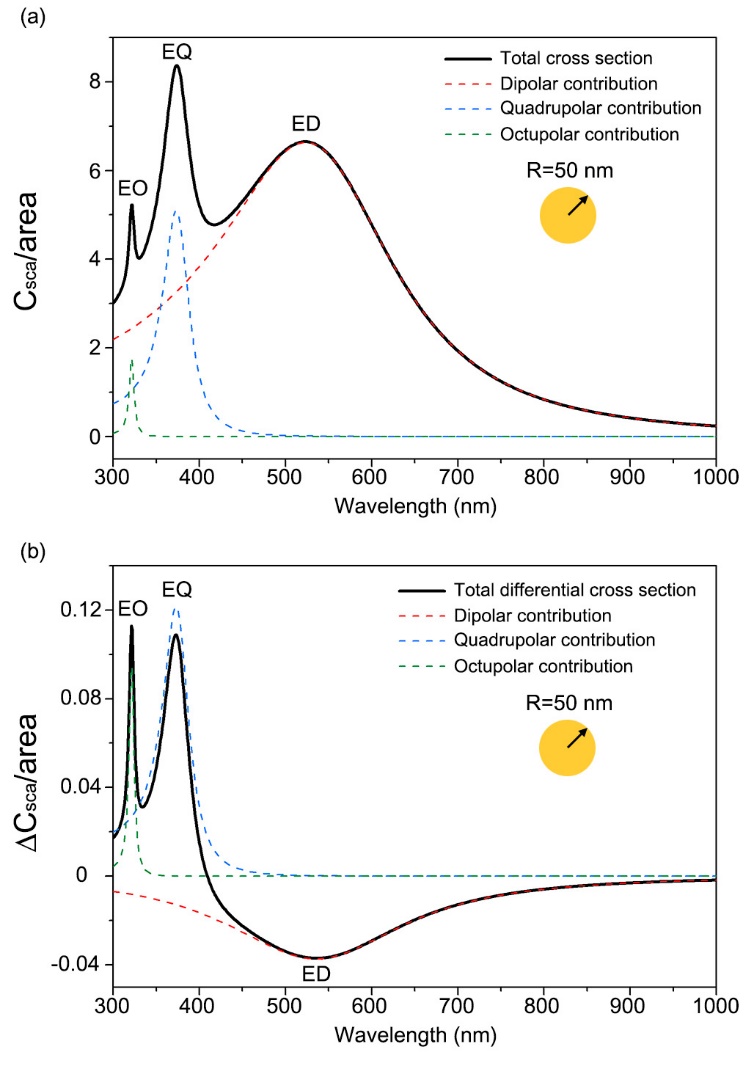


FIG. S2. (a) Scattering cross section of a Drude metal nanoparticle per particle area with $R$= 50 nm embedded in chiral molecule medium of refractive index $n$=1.5 and chirality parameter $\kappa$=0.01. Solid line (black) is the total scattering cross sections, and dashed lines (red, blue, and green) are electric dipolar (ED), quadrupolar (EQ), and octupolar (EO) contribution to the total scattering cross section, respectively. (b) Corresponding circular differential Scattering cross section per particle area of the Drude metal nanoparticle.

In the main text, the electric permittivity of gold is taken from the tabulated literature data^3^. Here, we instead use the Drude model for optical constants of metals. By the Drude model, permittivity of metals is given by

$\varepsilon\left(\omega\right)=1-\frac{\omega_{p}^{2}}{\omega^{2}+i\Gamma\omega}$,

where $\omega_{p}$ is the bulk plasma frequency, and $\Gamma$ is the damping term. In Figure S2, we use $\omega_{p}=13.8\times10^{15}\text{ }s^{-1}$ and $\Gamma=1.075\times10^{14}\text{ }s^{-1}$ as the fitting parameters^4^. The Drude model describes metals as the homogeneous free electron gas. However, real metals such as gold experience the effect of bound electrons, so-called interband transitions, in short wavelengths near blue^2,4^. The interband transitions of real metals result in stronger imaginary part of permittivity for real metals in comparison with the result of the Drude model. Real part of permittivity for real metals also can be positive due to the interband transition. When we compare Figure S2a&b (Drude metal) with Figure 2b&e (gold), we find the effect of the interband transition on CDMS. In Figure S2a&b (Drude metal), we can clearly find EQ and EO peaks in wavelengths shorter than ~400 nm because Drude metals lack loss from the interband transitions at short wavelengths. In Figure 2b&e (gold), we cannot find EQ and EO peaks because real metals experience severe loss from the interband transitions of bound electrons. We can conclude that peaks at 530 nm in Figure 2b ($C_{abs}/\text{area}$of a gold nanoparticle) and Figure 2e ($\Delta C_{abs}/\text{area}$of a gold nanoparticle) are originated from the interband transition of gold.

1. **Sensitivity of multi-resonances in high-index dielectric nanoparticles**


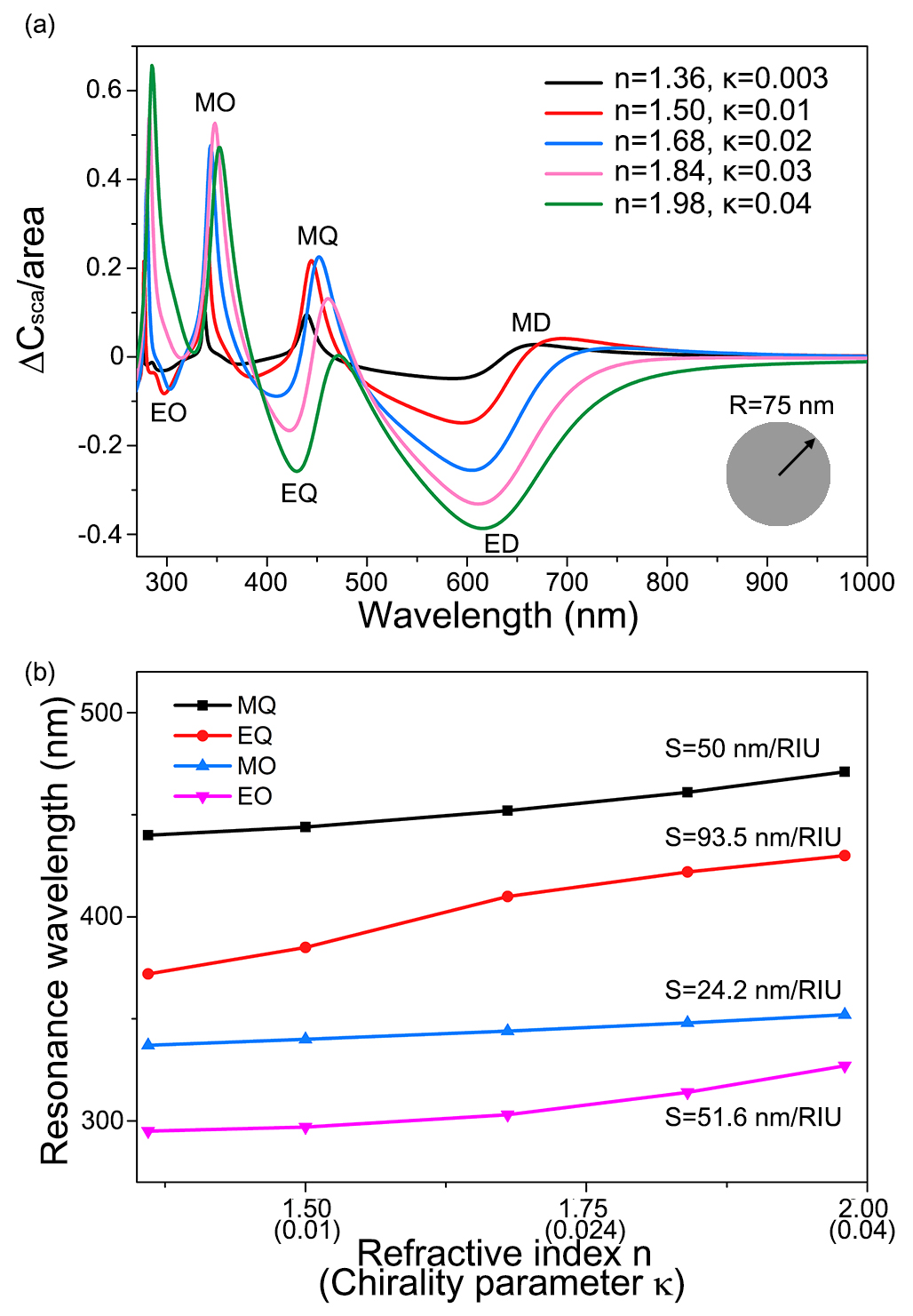


FIG. S3. (a) Circular differential scattering cross section of a high-index dielectric nanoparticle ($n_{1}$=4) whose radius $R$ is 75 nm, corresponding to changes in $n$ and $\kappa$. (b) Shifts in resonance wavelengths per refractive index unit (RIU) for higher order resonances. MQ, EQ, MO, and EO correspond to magnetic quadrupole, electric quadrupole, magnetic octupole, and electric octupole, respectively.

We characterize sensitivity (resonance shifts per refractive index unit, nm/RIU) of resonances in a high-index dielectric nanoparticle. Changes in refractive index $n$ and chirality parameter $\kappa$ of the surrounding chiral medium follow the equations, $\varepsilon=\varepsilon_{0}+N\alpha$ and $\kappa=NG$, as in the main text. Interestingly, sensitivity of resonances in a dielectric nanoparticle are poor in comparison with resonance in a plasmonic metal nanoparticle of the same size4 (see Fig. N in the main text). In a dielectric nanoparticle, resonances arises from whispering gallery modes (WGMs) which are standing modes inside the dielectric nanoparticle. Inside the dielectric nanoparticle, their resonance wavelengths mainly depend on refractive index $n_{1}$ and radius $R$ of nanoparticles due to their standing mode nature. This fact leads to poor sensitivity in a dielectric nanoparticles.

1. **Refractive index and chirality parameters in the weak dispersion region.**


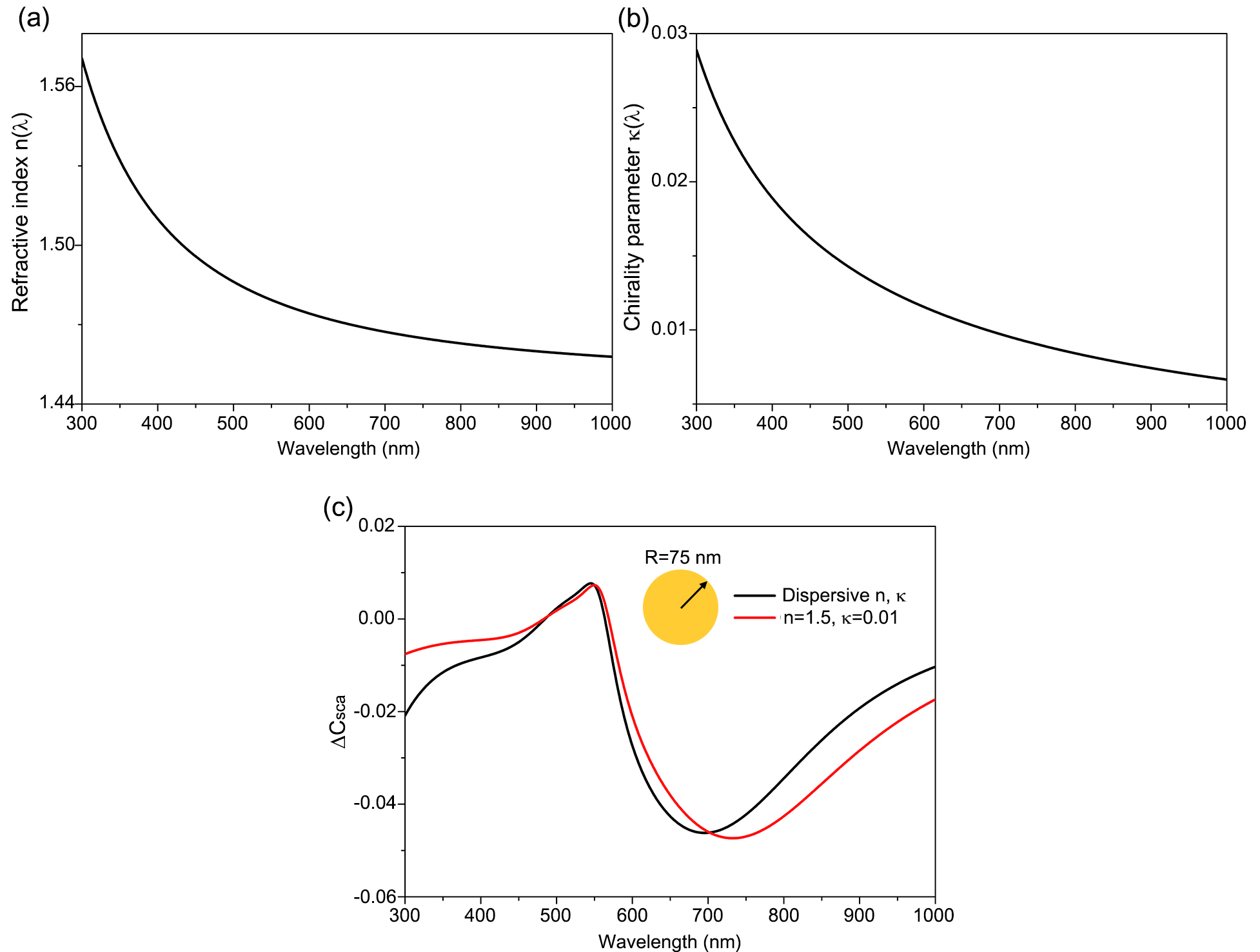


FIG. S4. (a) Refractive index and (b) chirality parameter of the weakly dispersive surrounding chiral medium. Dispersion follows Sellmeier equation (Eq. and ), and fitting parameters are $C_{1}$=1.0, $A_{1}$=6.5 nm, and $\lambda_{1}=150$ nm. (c) Circular differential scattering cross section per particle area of a gold nanoparticle with radius 75 nm. The same gold nanoparticles are immersed in dispersive medium with $n\left(\lambda\right)$ and $\kappa\left(\lambda\right)$ given by Fig. S4a&b (black line), and non-dispersive medium with $n$=1.5 and $\kappa$=0.01 (red line).

For weakly dispersive chiral molecules, the wavelength dependence of refractive index $n$ and chirality parameters $\kappa$ is described by Sellmeier equation^5^. The expressions of refractive index $n\left(\lambda\right)$ and chirality parameters $\kappa\left(\lambda\right)$ are^5^

$$n\left(\lambda\right)=\sqrt{1+\sum_{j} \frac{C_{j}\lambda^{2}}{\lambda^{2}-\lambda_{j}^{2}}}$$

$$\kappa\left(\lambda\right)=\sum_{j} \frac{A_{j}\lambda}{\lambda^{2}-\lambda_{j}^{2}}$$

where $C_{j}$ and $A_{j}$ are constants, and $\lambda_{j}$ is the absorption wavelength. The imaginary parts of refractive index $n\left(\lambda\right)$ and chirality parameters $\kappa\left(\lambda\right)$ are negligible in the weak dispersive molecules.

In Fig. S4, we calculate CDMS cross section of a gold nanoparticles immersed in weakly dispersive chiral molecules whose optical properties are described by Sellmeier equation (Eq. and ) In Fig. S4c, a gold nanoparticle immersed in weakly dispersive medium (black line) displays much stronger negative values in shorter wavelength near ultraviolet (UV) in comparison with the same gold nanoparticle immersed in non-dispersive medium with $n$=1.5 and $\kappa$=0.01 (red line).

**References**

1. Bohren, C. F. & Huffman, D. R. *Absorption and Scattering of Light by Small Particles*. (Wiley-VCH, 2012).

2. Quinten, M. *Optical Properties of Nanoparticle Systems*. (Wiley-VCH Verlag GmbH & Co. KGaA, 2011). doi:10.1002/9783527633135

3. Johnson, P. B. & Christy, R. W. Optical Constants of the Noble Metals. *Phys. Rev. B* **6,** 4370–4379 (1972).

4. Novotny, L. & Hecht, B. *Principles of Nano-Optics*. (Cambridge University Press, 2012).

5. Barron, L. D. *Molecular Light Scattering and Optical Activity*. (Cambridge University Press, 2004).
